# Introducing two new Main Editors of **IUCrJ**

**DOI:** 10.1107/S2052252521013129

**Published:** 2021-12-16

**Authors:** Peter R. Strickland

**Affiliations:** a International Union of Crystallography, 5 Abbey Square, Chester, CH1 2HU, United Kingdom

**Keywords:** FELs, electron crystallography

## Abstract

Henry Chapman and Xiaodong Zou have recently been appointed as Main Editors of **IUCrJ** .

Henry Chapman of the Center for Free-Electron Laser Science, Deutsches Elektronen-Synchrotron DESY, and Xiaodong Zou of the Department of Materials and Environmental Chemistry, Stockholm University, have recently been appointed as Main Editors of **IUCrJ** . Henry Chapman, who was already a Co-editor of **IUCrJ** , will succeed the late John Spence in leading the journal section on *Physics and Free Electron Laser Science and Technology*. Xiaodong Zou will lead a new section on *Electron Crystallography*, which will act as a home within the IUCr journals for high-quality, high-impact papers in this field. We are assembling a team of Co-editors to work with Xiaodong Zou and details of this section will be provided in the next issue of **IUCrJ** .

*Henry Chapman* is a physicist who is a world leader in exploiting modern high-brightness X-ray sources for high-resolution structure determination and imaging of soft matter or biological systems. His work in developing experimental methods and analysis for coherent diffractive imaging of non-periodic objects includes the first experimental demonstration of X-ray ptychography, as well as his pioneering work in serial femtosecond crystallography. His international status in these FELS fields also includes his association with multiple world-class high-brightness coherent X-ray source developments around the world. Henry completed his PhD in 1992 at The University of Melbourne, Australia, for which he was awarded the Bragg Gold Medal from the Australian Institute of Physics. He explored lensless X-ray imaging at Stony Brook University (NY, USA), and first demonstrated X-ray flash imaging while at Lawrence Livermore National Laboratory in California where he also contributed to the development of extreme ultraviolet lithography. He joined DESY and the University in Hamburg in 2007 as a founding director of the Center for Free-Electron Laser Science. He was awarded the Leibniz Prize of the German Research Foundation (DFG), the Roentgen Medal, and an honorary doctorate of Uppsala University, and is a Fellow of the Royal Society (FRS).

*Xiaodong Zou* is a leading expert in electron crystallography and porous materials. Her research interests have been developments of electron crystallographic methods and design of novel porous materials. She is one of the pioneers in establishing electron crystallography as an important technique for accurate atomic structure determination of unknown 3D crystals. Her group has demonstrated the power of electron crystallography in studying complex structures including zeolites, metal-organic frameworks, pharmaceuticals and proteins. Professor Zou has also made key contributions in design, synthesis and applications of novel zeolites and metal-organic frameworks. Xiaodong is a full professor in structural chemistry and deputy head of the Department of Materials and Environmental Chemistry, Stockholm University. She received her BSc in Physics at Peking University in 1984, and PhD in structural chemistry at Stockholm University in 1995. One of her main research interests is method development for accurate atomic structure determination of nano-sized crystals by electron crystallography. Her group has solved a number of complex structures of zeolites and mesoporous crystals by transmission electron microscopy. She is also working on synthesis, structure determination, topology analysis and applications of inorganic open-framework materials and metal-organic frameworks. She has received several prestigious awards given by the Royal Swedish Academy of Sciences. She is an elected member of the Royal Swedish Academy of Sciences (KVA), member of the Royal Swedish Academy of Engineering Sciences (IVA), Fellow of the Royal Chemical Society (FRCS) and council member of the International Zeolite Association.

